# USP38: an important regulatory factor in tumor malignant progression

**DOI:** 10.3389/fimmu.2025.1612723

**Published:** 2025-08-27

**Authors:** Junyan Li, Jinghua Zhong, Jianming Ye, Yi Xiang, Qiang Yi, Gangfeng Zhu, Shifan Deng, Xiangcai Wang

**Affiliations:** ^1^ The First Clinical Medical College, Gannan Medical University, Ganzhou, Jiangxi, China; ^2^ The Oncology Department of the First Affiliated Hospital of Gannan Medical University, Ganzhou, Jiangxi, China; ^3^ Jiangxi Clinical Research Center for Cancer, Ganzhou, Jiangxi, China

**Keywords:** USP, USP38, deubiquitination, expression, mechanism, malignancy

## Abstract

Ubiquitin-Specific Protease 38 (USP38), a member of the deubiquitinating enzyme (DUB) family, exhibits a complex and context-dependent role in cancer progression. This review summarizes current research on USP38, highlighting its dual functionality as both an oncogene and a tumor suppressor in various malignancies. We detail the structural characteristics of USP38, its differential expression patterns across cancer types, and its impact on key cellular processes including proliferation, migration, invasion, and apoptosis. Mechanistically, USP38 regulates the stability and activity of crucial proteins involved in tumorigenesis, such as HDAC1/3, LSD1, KLF5, METTL14, c-Myc, and HIF-1α, as well as influencing signaling pathways like JAK2/STAT3. The intricate interplay and, in some instances feedback loops, between USP38 and its targets underscore its multifaceted role. Finally, we discuss the potential of USP38 as a therapeutic target, the challenges in developing specific inhibitors, and future research directions to fully elucidate its complex biology and clinical implications.

## 1 Introduction

Cancer remains the primary threat to human life and health, with patients facing not only the substantial physical and mental suffering caused by tumors, but also the considerable economic burden and a severely compromised quality of life (1–6). Throughout the treatment process for cancer patients, individual differences, tumor heterogeneity, and the limitations of treatment regimens contribute to a decreased sensitivity of tumors to drugs, resulting in suboptimal treatment outcomes and poor prognosis (7). Therefore, identifying new targets for effective cancer treatment has become an urgent priority. Exploring the regulation of tumor gene-encoded protein homeostasis has become a research hotspot, as the regulation of abnormal protein expression involves numerous molecules, providing potential for precision medicine in cancer therapy (8). Given that deubiquitinating activity can effectively regulate the protein stability of key tumor genes, USP enzymes are considered to be a class of promising cancer biomarkers.

The primary function of deubiquitinating enzymes (DUBs) is to remove ubiquitin chains from target proteins to prevent their degradation by the proteasome (9, 10). DUBs consist of five families: ubiquitin-specific proteases (USPs), ubiquitin C-terminal hydrolases (UCHs), Machado-Joseph disease protein domain proteases (MJDs), ovarian tumor proteases (OTUs), and JAMM motif proteases (11). Among these, USPs have been shown to have more than 50 family members, each possessing a conserved USP domain. Through the catalytic site within this domain, USPs can bind to the ubiquitin of specific proteins and exert their deubiquitinating function, thereby maintaining protein stability. This post-translational modification is essential for the maintenance of protein homeostasis (12, 13). Furthermore, abnormal expression of USPs has been linked to various diseases, including inflammatory diseases (14), metabolic disorders (15), and cardiovascular diseases (16). By directly or indirectly regulating the protein expression levels of pathway-related target molecules, USPs can influence the onset and progression of these diseases. Therefore, an in-depth exploration of the regulatory mechanisms of USPs in human diseases is of great clinical significance.

USP38, a protein-coding gene located on chromosome 4q31.21 with 38,958 nucleotides (https://www.ncbi.nlm.nih.gov/gene/84640), encodes a protein known as Ubiquitin carboxyl-terminal hydrolase 38 (UCH38), consisting of 1,042 amino acid residues and exhibiting deubiquitinating activity. USP38 was first mentioned by Mathieu Ferron et al. in 2006 during their investigation of the mouse Inpp4b gene. Subsequently (17), in 2011, Tomomitsu Hirota and colleagues identified USP38 as part of a genetic susceptibility region shared with the GAB1 gene, which is associated with susceptibility to adult asthma in Japan (18). In recent years, although studies have explored the regulatory roles of USP38 in various diseases, including cardiovascular diseases (19, 20), asthma (21), and inflammatory responses (22), most research has focused on its role in malignant tumors. A growing body of evidence suggests that USP38 not only exhibits abnormal expression in various malignant cancers but is also closely involved in their initiation and progression, including gastric cancer, bladder cancer, and colorectal cancer, highlighting its crucial role in regulating the malignant progression of tumors.

## 2 The genetic and protein information of USP38

**Figure 1:**
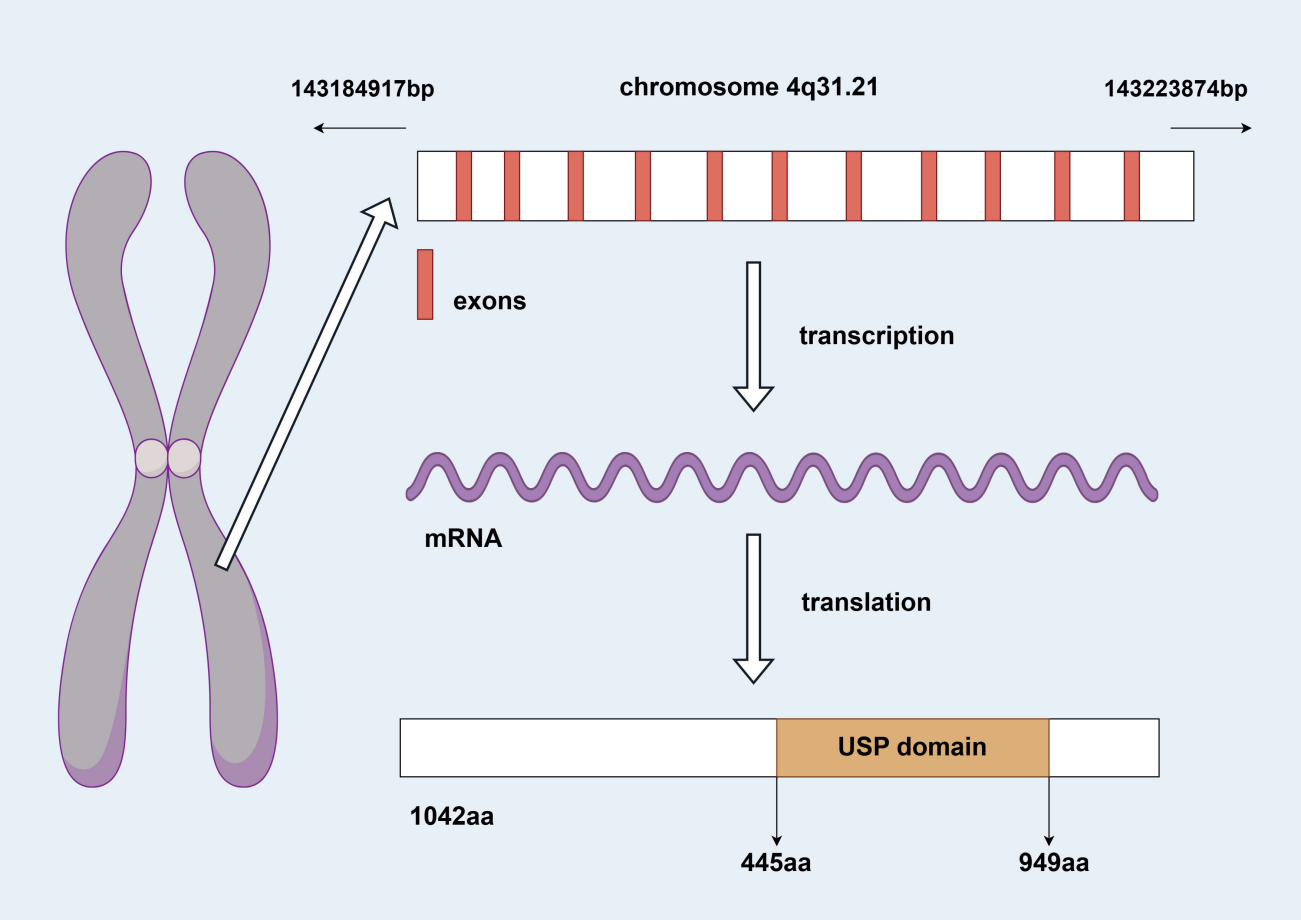
Genomic information of USP38 (by Figdraw 2.0).

**Figure 2:**
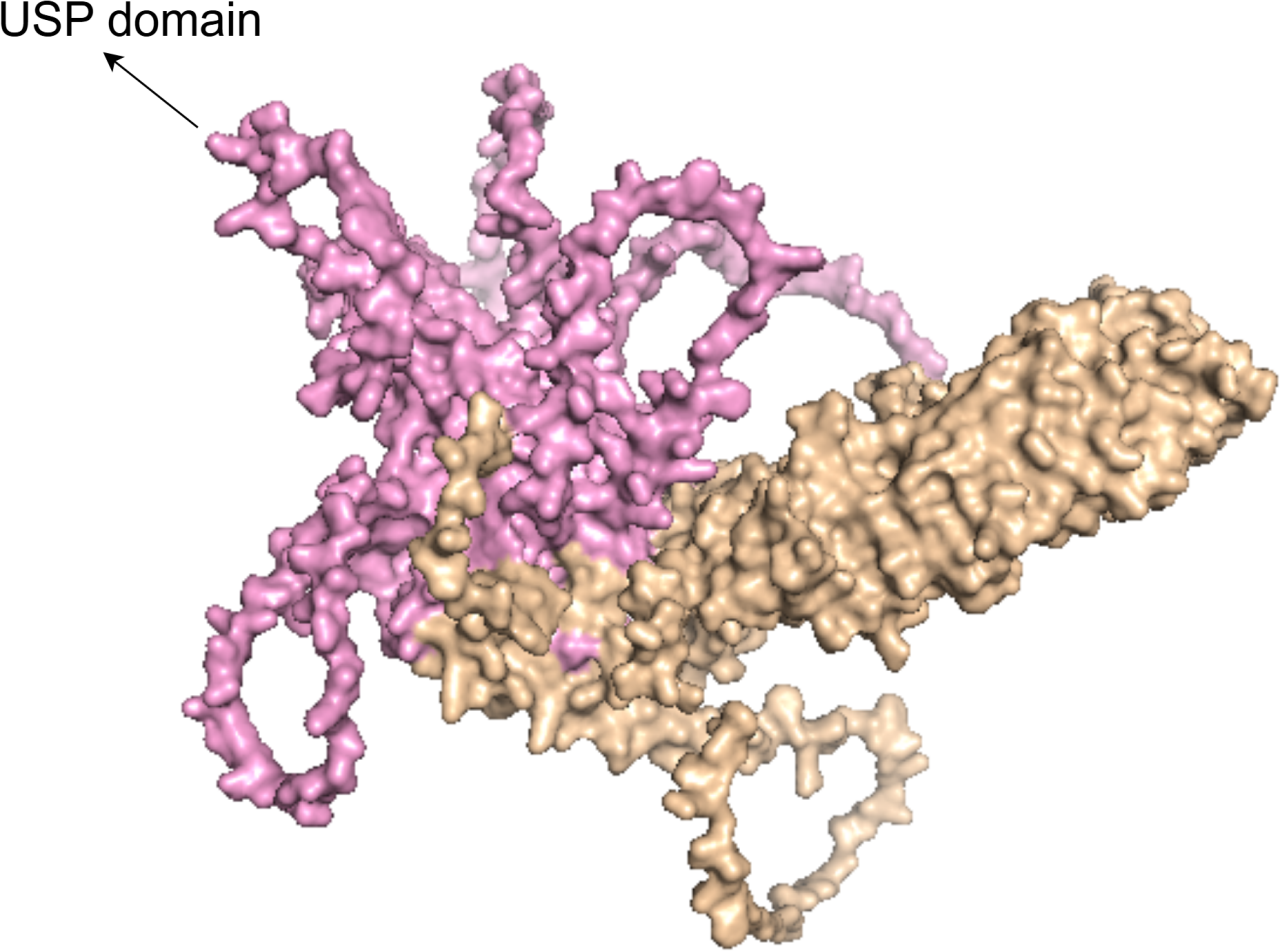
The figure shows the 3D structural model of the USP38 protein, with the pink region representing the USP domain.

The USP38 gene is located on the q31.21 region of chromosome 4, spanning the genomic DNA positions from 143,184,917 to 143,223,874, with a total length of 38,958 nucleotides. This gene consists of 11 exons and 10 introns (as shown in **Figure 1**). The two adjacent genes of USP38, LOC100287014 and GAB1, are located 138,842 nucleotides and 113,002 nucleotides away from USP38, respectively (https://www.ncbi.nlm.nih.gov/gene/84640). The protein translated from USP38 consists of 1,042 amino acids, with the region between amino acids 445 and 949 falling within the USP domain, which is likely the functional site responsible for the deubiquitinating activity of USP38 (https://www.uniprot.org/uniprotkb/Q8NB14/entry) (as shown in **Figure 2**). Additionally, it is noteworthy that studies have shown that USP35 shares a similar amino acid sequence with USP38, suggesting that they may have comparable biological functions.

## 3 Clinical sample study of USP38

In lung adenocarcinoma, USP38 is abnormally overexpressed, and its elevated expression level is closely associated with lymph node metastasis and TNM staging. Kaplan-Meier analysis indicates that high USP38 expression is linked to poor prognosis. Additionally, Cox regression analysis suggests that the abnormal expression of USP38 can serve as an independent risk factor for the outcomes of LUAD patients. In gastric cancer, analysis of the cancer genome atlas (TCGA) database reveals that USP38 is highly expressed in gastric cancer tissues compared to adjacent non-cancerous gastric tissues. Moreover, overexpression of USP38 is positively correlated with tumor grade and stage. These findings were further validated by RT-qPCR and immunohistochemistry (IHC). In esophageal squamous cell carcinoma, GEPIA database analysis shows that USP38 expression in esophageal squamous cell carcinoma (ESCC) tissues is significantly higher than in normal tissues, and its overexpression is significantly negatively correlated with relapse-free survival. Interestingly, unlike the aforementioned malignancies, the expression level of USP38 is significantly reduced in colorectal cancer and clear cell renal carcinoma tissues. In colorectal cancer, further analysis of the TCGA dataset reveals a negative correlation between USP38 expression and tumor malignancy (As shown in the **Table 1**).

**Table 1:** USP38 in the clinical pathological features of various tumors.

Cancer type	Number of cases	Expression	Prognosis	Clinicopathological characteristics	Reference
CRC	30	low	good	Malignancy grade	(32)
KIRC	75	Low	/	/	(37)
LUAD	98	high	poor	TNM stage, Lymph node metastasis	(98)
GC	18	high	poor	Tumor stage, Tumor grade	(93)
ESCC	/	high	poor	recurrence-free survival	(104)

These findings suggest that USP38 may have dual regulatory roles in malignant tumors, acting both as an oncogene and as a tumor suppressor. The potential mechanisms by which USP38 regulates tumor progression are complex, and likely depend on the specific tumor microenvironment.

## 4 Cellular studies of USP38

**Figure 3:**
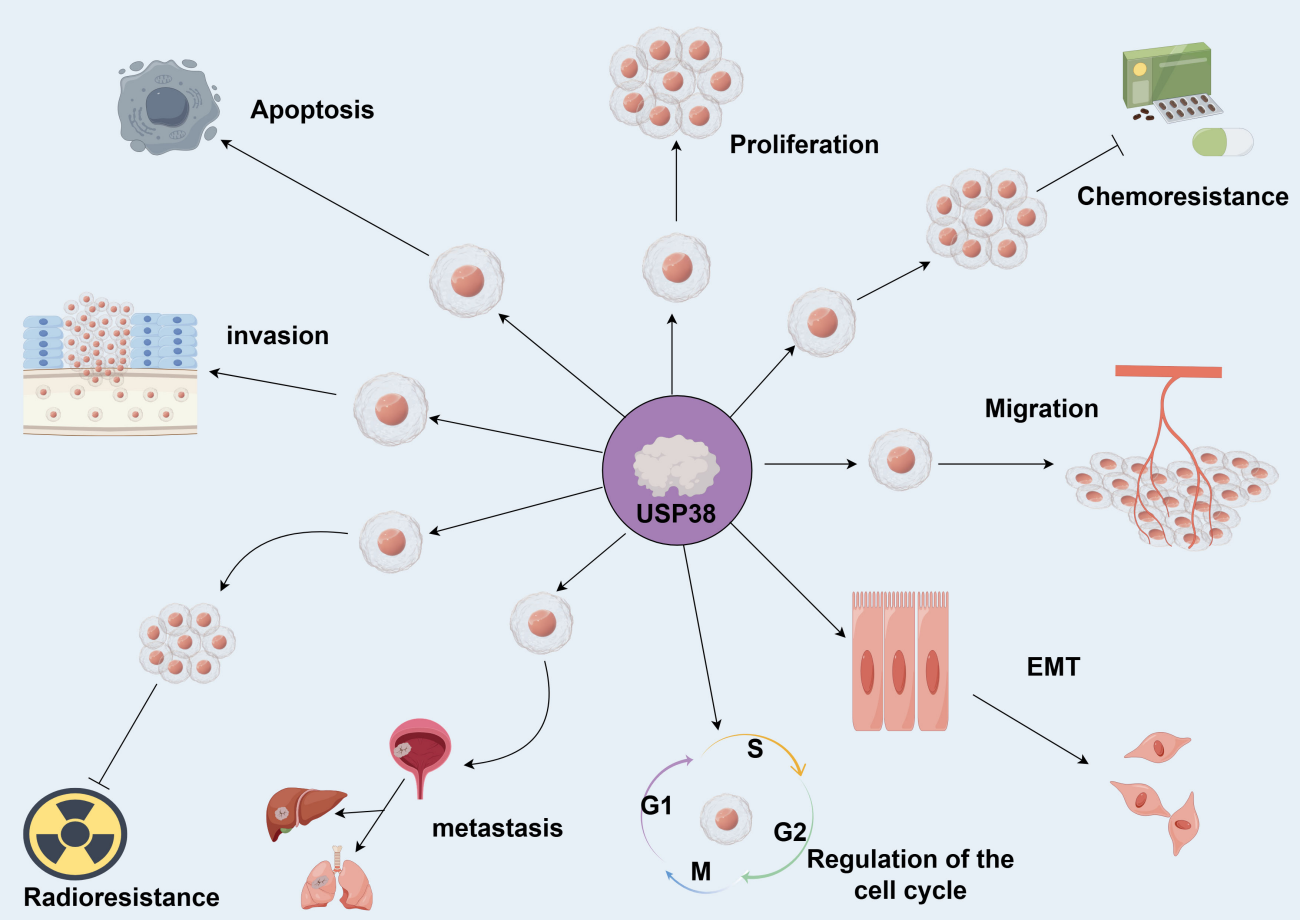
USP38 is capable of regulating various biological functions of tumors. Promotes →; Inhibits →. (by Figdraw 2.0).

USP38 can regulate various biological behaviors of tumors and influence tumor initiation and progression (as shown in **Figure 3**). In different cancer cell lines, the expression of USP38 exerts different regulatory effects on tumor cell growth. For instance, in esophageal squamous cell carcinoma, glioma, lung adenocarcinoma, and gastric cancer cell lines, knockdown of USP38 effectively inhibits tumor cell proliferation, while overexpression can reverse this process. However, in clear cell renal carcinoma and bladder cancer cell lines, knockdown of USP38 actually promotes tumor cell proliferation, indicating that the expression level of USP38 varies across different tumor cell lines. Notably, unlike the aforementioned tumor cell lines, the regulatory effect of USP38 expression on cell growth and proliferation in colorectal cancer cell lines shows contradictory results in different experiments. In the study by Wei Zhan et al., upregulation of USP38 inhibited cell growth and proliferation, while in the study by Wenbin Liu et al., they found that overexpression of USP38 in wild-type LSD1 HCT116 cells promoted cell proliferation. There are many factors contributing to this differential phenomenon, including differences in the cell lines themselves, cell culture conditions and environments, the threshold level of USP38 expression, and the complex regulation of signaling pathways, all of which require further experiments for clarification (As shown in **Table 2**).

**Table 2:** The biological functions and mechanisms of USP38 in tumors.

Cancer	Expression	Functional	Related gene	Role	Reference
CRC	Downregulated	Inhibited cell proliferation and chemoresistance	HDAC3	Tumor Suppressor Gene	(32)
	Downregulated	Inhibited cell proliferation, migration, and invasion	HMX3	Tumor Suppressor Gene	(49)
KIRC	Downregulated	Inhibited the sensitivity of tumor cells to ionizing radiation (IR).	HDAC1	Tumor Suppressor Gene	(37)
BCa	Downregulated	Inhibited migration, invasion, metastasis, and epithelial–mesenchymal transition (EMT).	miR-3165/METTL14/YTHDF2	Tumor Suppressor Gene	(110)
Glioma	Downregulated	Inhibited cell proliferation, invasion, migration, metastasis and promoted apoptosis	STAT3	Tumor Suppressor Gene	(76)
NSCLC	Upregulated	Inhibited apoptosis	HIF1α	oncogene	(61)
LUAD	Upregulated	Promoted cell proliferation	KLF5	oncogene	(98)
GC	Upregulated	Promoted cell proliferation, migration, cell cycle, and inhibited apoptosis.	FASN	oncogene	(93)
ESCC	Upregulated	Promoted cell proliferation	ADAR	oncogene	(104)

The method of predicting tumor prognosis by evaluating the levels of biomarkers in serum has gained increasing attention. Physicians can predict disease progression, treatment efficacy, and survival expectations by assessing various indicators, thereby continuously optimizing treatment strategies to improve patient survival rates and quality of life (23, 24). In 2017, Dora Londra and colleagues discovered the feasibility of predicting prostate cancer prognosis by detecting the methylation level of the USP44 promoter in serum. In their study, the researchers applied real-time methylation-specific PCR technology and found no detection of USP44 promoter methylation in the serum of healthy individuals (n=10) and early-stage prostate cancer patients (n=32). However, in patients with confirmed metastasis (n=39), 20 samples showed positive detection of USP44 promoter methylation, with a detection rate of 51.3% (25). This result suggests that USP44 can be detected in serum and used to predict tumor prognosis. Therefore, USP38, which belongs to the same family as USP44, could theoretically also be detected in serum. Notably, based on current research, we found that USP38 mainly exerts its regulatory function during the post-translational modification phase, rather than at the transcriptional stage. Therefore, unlike USP44, which is detected through the methylation level of the promoter, the substance for liquid biopsy should be the USP38 protein molecule. In the future, we plan to further investigate the regulatory mechanisms of USP38 and develop a highly sensitive and specific serum USP38 detection technology, which will be validated by assessing USP38 protein levels in the serum of healthy individuals and patients with different tumor stages.

## 5 Animal studies of USP38

Related studies have found that in xenograft models of lung adenocarcinoma and esophageal squamous cell carcinoma, the tumor volume and size in the USP38 knockdown group were significantly smaller than in the control group. In contrast, in gastric cancer and lung adenocarcinoma models, overexpression of USP38 promoted an increase in tumor volume and weight. Additionally, it is noteworthy that, unlike the aforementioned cancer types, in colorectal cancer animal models, overexpression of USP38 resulted in smaller tumor volume and weight compared to the control group (As shown in **Table 3**), suggesting that the regulatory role of USP38 may vary across different malignant tumors due to tissue-specific differences.

**Table 3:** Effects of USP38 on growth and metastasis of cancer xenografts.

Cancer type	Animal models	Function	References
CRC	6 weeks-old-female BALB/c nude mice	↑↑USP38: ↓tumor growth ↓↓USP38: ↑tumor growth	(32)
LUAD	6 weeks-old-female BALB/c nude mice	↑↑USP38: ↑tumor growth ↓↓USP38: ↓tumor growth	(98)
GC	6 weeks-old-male BALB/c nude mice	↑↑USP38: ↑tumor growth	(93)
ESCC	5 weeks-old-female BALB/c nude mice	↓↓USP38: ↓tumor growth	(104)

## 6 The biological function and mechanism of USP38

**Figure 4:**
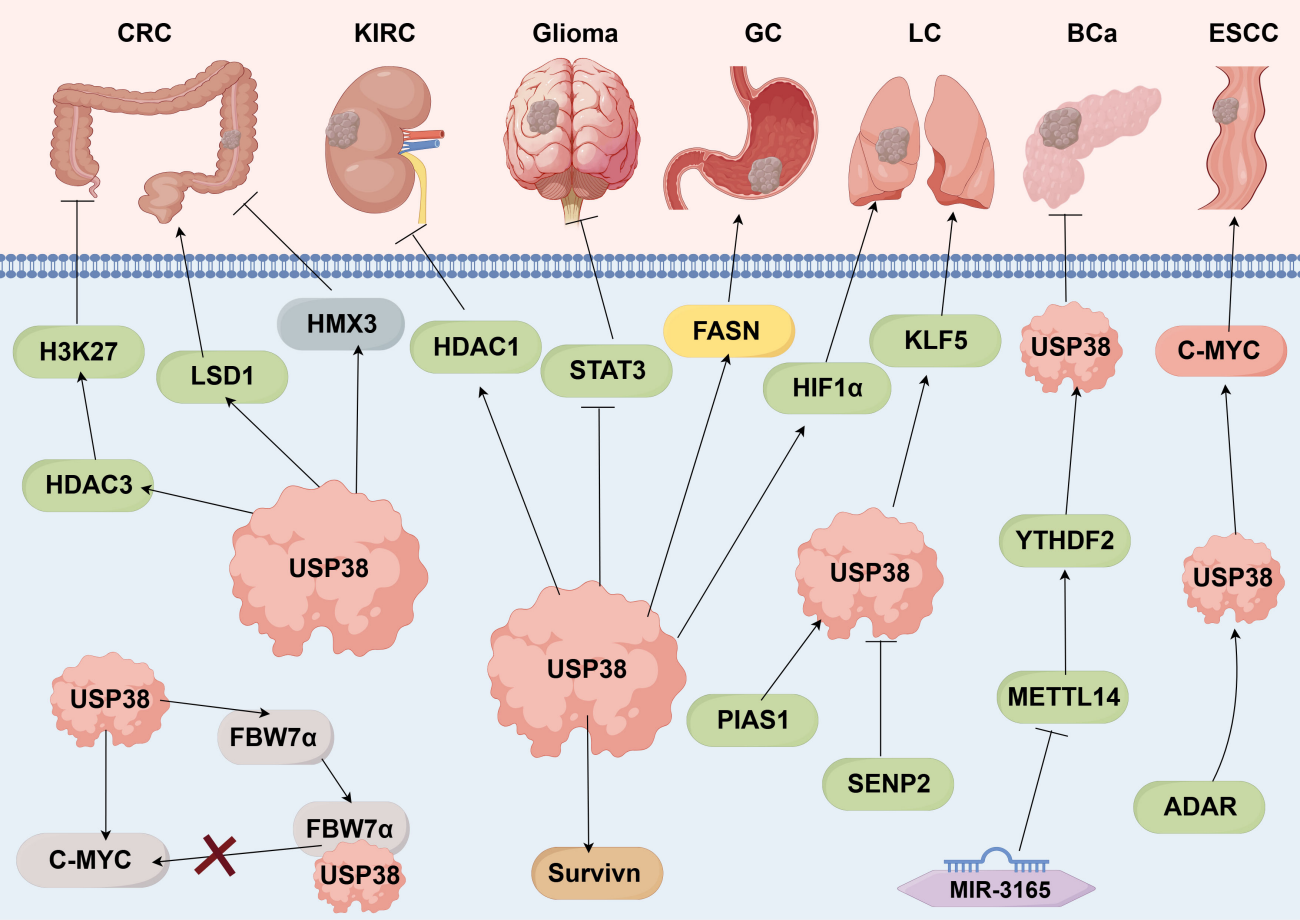
USP38-related signaling pathways. Promotes →; Inhibits → (by Figdraw 2.0).

Recent studies have revealed that USP38 modulates critical molecules in multiple signaling pathways, such as JAK2/STAT3, miR-3165/METTL14/YTHDF2, through deubiquitination, thereby exerting an indirect influence on the malignant progression of tumors (as shown in **Figure 4**).

### 6.1 downstream regulatory mechanisms

#### 6.1.1 HDAC3

HDAC3, as a member of the Class I HDAC family, functions by removing acetylation marks from histone proteins in the promoter regions of downstream molecules, thereby suppressing the expression of target genes (26). In malignancies, HDAC3 regulates multiple signaling pathways, influencing tumor initiation and progression (27, 28). In gastric cancer research, Wenjing Zhao and colleagues discovered that HDAC3 is recruited by LINC00355 and binds to the promoter region of TP53INP1. By removing acetylation marks from histone H3, HDAC3 inhibits the transcription of TP53INP1 (29). TP53INP1 has been identified as a tumor suppressor gene and, as a target gene of P53, it effectively inhibits tumor cell proliferation and promotes cell apoptosis (30, 31). As a multifunctional regulator, HDAC3 activates multiple signaling pathways by deacetylating the promoter regions of target molecules, indirectly influencing the progression of malignant tumors. Given its importance, further exploration of the regulatory mechanisms affecting HDAC3 expression is crucial. In the study of USP38 regulation of HDAC3 ubiquitination, Wei Zhan et al. found through Western blotting and qPCR experiments that downregulation of USP38 expression inhibited the transcriptional activity of tumor stemness-related genes such as CD133 and SOX2 (32). However, current research generally suggests that USP38 regulates the ubiquitination of target proteins at the post-translational modification level, which implies that USP38 may indirectly influence the expression of tumor stemness-related genes by regulating downstream molecules. Subsequent IP and ChIP experiments confirmed this hypothesis. USP38, through its deubiquitinating activity, removes the K63 polyubiquitin chain at the K121 site of HDAC3, enhancing HDAC3’s deacetylation of H3K27 in the promoter regions of tumor stemness-related genes, thereby effectively regulating tumor stem cell properties.

USP38 is one of the few deubiquitinating enzymes in the USP family that has been shown to effectively regulate HDAC3 activity. Although current research is primarily focused on colorectal cancer, the downstream target HDAC3 has been shown to play a regulatory role in various malignancies. Based on the protein interaction between USP38 and HDAC3, we believe that the regulation and activation of the USP38/HDAC3 signaling axis may be common across multiple tumor types. Furthermore, USP38’s regulation of HDAC3 does not affect its protein expression levels, suggesting that the amino acid site regulating HDAC3 protein stability is not at K121, and may be regulated at other sites by members of the USP family. This requires further experimental screening.

#### 6.1.2 HDAC1

HDAC1 and HDAC3, being members of the same family, share functional similarities, including their deacetylase activity (33, 34). However, the signaling pathways they regulate differ significantly, particularly in malignancies (35, 36). Research has shown that USP38 not only regulates the deacetylase activity of HDAC3 but also affects the ubiquitination levels of HDAC1 and its deacetylase activity. In studies on genotoxic stress response, Yongfeng Yang et al. found that USP38 influences HDAC1’s deacetylase activity by removing K63-linked polyubiquitin chains, thereby impacting DNA damage repair processes (37). It is noteworthy that USP38’s regulation of HDAC1 and HDAC3 is similar: by deubiquitinating both enzymes, USP38 enhances their deacetylase activity, yet this effect does not alter their overall protein expression levels. Since K63-linked polyubiquitin chains primarily influence the functional activity of target proteins, while K48 chains mainly affect protein degradation, the amino acid residues responsible for regulating HDAC1 protein stability and deacetylase activity may not be the same. It is likely that the amino acid residues that maintain HDAC1 stability are attached to K48-linked polyubiquitin chains. This hypothesis has been validated by existing research. Yuhong Ma et al. demonstrated that ABIN1 maintains HDAC1 protein stability by removing K48-linked polyubiquitin chains from HDAC1 (38).

In conclusion, unlike USP38’s regulation of other target proteins’ ubiquitination to affect their stability, USP38’s regulation of HDAC1 ubiquitination directly enhances its deacetylase activity, highlighting the diversity of USP38’s regulatory functions. Furthermore, USP38’s regulation of HDAC1 enzyme activity indirectly participates in the DNA damage repair process, providing new insights for clinical cancer treatment and prevention. In cancer screening, detecting USP38 expression levels can help assess a patient’s cancer risk. During tumor treatment, analyzing USP38 expression levels in cancer tissues can be used to evaluate the patient’s prognosis and inform the development of targeted treatment strategies.

#### 6.1.3 LSD1

LSD1, as a demethylase, regulates gene transcription activity by removing methylation marks from both histone and non-histone proteins, thereby influencing the expression levels of proteins encoded by specific genes (39, 40). In malignancies, LSD1 regulates multiple downstream signaling networks and indirectly affects various biological functions of malignant tumor cells by controlling the expression of tumor-associated genes. Studies have found that several members of the USP family regulate the expression of the protein encoded by LSD1.In breast cancer, the deubiquitinating function of USP7 enhances LSD1 protein stability, while CARM1 further enhances USP7’s positive regulation of LSD1 stability by dimethylating the arginine residue at position R838 of LSD1. The upregulated LSD1 then suppresses E-cadherin expression and activates vimentin expression, promoting breast cancer cell invasion and metastasis (41). Additionally, in glioma, Lei Yi et al. found that USP7 also binds to LSD1 to exert deubiquitinating effects, promoting tumor cell growth and proliferation, suggesting that USP7 regulates LSD1 expression across multiple tumor types (42). In osteosarcoma, Wei Liu et al. discovered that polyubiquitin chains attached to LSD1’s amino acid residues can be removed by USP22, thereby stabilizing LSD1. The stabilized LSD1 then demethylates histone H3 at the p21 promoter region, inhibiting the expression of p21 (43). As a tumor suppressor gene, the downregulation of p21 results in the suppression of apoptosis. Similar to USP7 and USP22, USP38 also maintains LSD1 protein stability by removing polyubiquitin chains from its amino acid residues (44). However, USP38 primarily relies on the cysteine residue (C454) in its USP domain to catalyze this process. Since the catalytic active sites of these three USPs are located at different positions, the amino acid residues targeted by USP38 on LSD1 may also differ, suggesting that USP38’s regulation of LSD1 is specific. Furthermore, given USP38’s positive regulatory effect on LSD1, the development of USP38/LSD1-targeted inhibitors could, in theory, effectively suppress the malignant progression of specific tumors. However, this hypothesis requires further experimental validation.

#### 6.1.4 HMX3

HMX3 belongs to the homeobox gene family. Previous studies have primarily focused on its role in the regulation of retinal and ear development (45, 46). However, recent research has identified that HMX3 also plays a role in regulating the malignant progression of tumors. In acute myeloid leukemia (AML), Stefan Nagel et al. found that HMX3 not only suppresses the expression of the eosinophil differentiation-related gene EPX but also promotes the abnormal activation of the ERK pathway, which in turn facilitates tumor cell growth and proliferation (47). Additionally, in one subtype of AML, MECOM-negative KMT2A::MLLT3 leukemia, Saioa Arza-Apalategi et al. discovered that HMX3 promotes leukemia cell proliferation by activating the expression of oncogenes such as E2F and MYC (48). These findings indicate that HMX3 has a positive promotive effect on certain tumors. However, in a subsequent study on colorectal cancer, Jun Wang et al. found that the overexpression of HMX3 inhibits tumor cell proliferation, suggesting that the regulatory role of HMX3 in tumors may be tissue-specific. To further investigate the mechanism of HMX3’s action, researchers conducted bioinformatics analysis to identify genes associated with HMX3. The results revealed that USP38 is correlated with HMX3. Subsequent immunoprecipitation and deubiquitination experiments demonstrated that the inhibitory effect of HMX3 on tumor cells is USP38-dependent: USP38’s deubiquitinating activity enhances HMX3 protein stability, thus strengthening its tumor-suppressive effect (49).

The positive regulatory effect of USP38 on HMX3 expression indicates that in colorectal cancer, USP38 can influence tumor cell growth by regulating multiple signaling pathways (such as the USP38/HDAC3 signaling axis and the USP38/LSD1 signaling axis). This highlights the complexity of USP38’s role in tumor regulation.

#### 6.1.5 c-Myc

c-Myc, as an important transcription factor, plays a key regulatory role in tumor proliferation, metabolism, apoptosis, and other processes (50). In osteosarcoma, Shizhe Li et al. found that c-Myc is involved in the regulation of the p53/c-Myc/Cyclin D1 signaling pathway. By directly acting on the Cyclin D1 promoter region, c-Myc promotes Cyclin D1 transcription, thereby influencing tumor cell proliferation (51). In hepatocellular carcinoma, Ruiqi Liu et al. discovered that c-Myc directly activates the transcriptional activity of glycolytic genes such as HK2, GLUT1, and LDHA, effectively promoting the glycolysis process in the tumor microenvironment (52). These findings suggest that c-Myc’s regulation of downstream target molecules can impact multiple tumor biological functions.

In the regulation of c-Myc gene expression, multiple molecules can regulate c-Myc protein expression, particularly at the post-translational modification level, where the protein stability of c-Myc can be regulated by USP family members. In bladder cancer, Xia Zhang et al. found that USP1 directly binds to c-Myc and removes the ubiquitin tags on c-Myc through its deubiquitinating function (53). In pancreatic cancer, Jichun Gu et al. discovered that USP7 also directly interacts with c-Myc to promote its protein stability (54). In the aforementioned studies, USP1 and USP7 directly regulate c-Myc, while other research has shown that USP38 can indirectly regulate c-Myc expression. Zhijun Xu et al. found that phosphorylated c-Myc can be recognized and bound by FBW7α, leading to its ubiquitination and degradation. USP38, however, can inhibit this process by binding to FBW7α, thereby indirectly maintaining c-Myc protein stability (55).

Unlike USP38’s direct interactions with other molecules, its regulation of c-Myc is indirect, reflecting its diversity in regulatory functions. Furthermore, USP38 mutants with lost catalytic activity are still able to bind to FBW7α, demonstrating that USP38’s regulation of other molecules is not solely dependent on its deubiquitinating activity, highlighting its functional diversity. However, the current research on the structure and function of USP38 remains insufficient. Further exploration of the different functional domains in the USP38 amino acid sequence will not only deepen our understanding of its multifunctionality but also provide a theoretical foundation for the development of USP38 inhibitors.

#### 6.1.6 HIF-1α

HIF-1α is a transcription factor that is activated under hypoxic conditions. It binds with HIF-1β in the nucleus to form the HIF-1 complex, which promotes the transcription of target genes by recognizing hypoxia response elements (HREs) in their promoter regions. Under normal physiological conditions, HIF-1α expression levels are very low, but it still regulates the expression of corresponding genes to help cells adapt to the hypoxic microenvironment. In malignant tumors, HIF-1α, in response to low oxygen levels, plays a critical role in multiple biological processes, including tumor cell migration and invasion, tumor angiogenesis, and immune evasion (56). In research on tumor immune evasion, Christopher M. Bailey et al. found that HIF-1α directly regulates the transcription of PD-L1 by acting on the core hypoxia response element (HRE) in the PD-L1 promoter. Furthermore, aberrant expression of HIF-1α not only promotes PD-L1 expression but also suppresses the function of tumor-infiltrating lymphocytes (TILs), ultimately activating tumor immune evasion (57).

The expression level of HIF-1α protein is primarily influenced by oxygen levels. Under normal or high oxygen conditions, the proline residues of HIF-1α are hydroxylated by prolyl hydroxylases, and the subsequently activated ubiquitin ligase system accelerates the degradation of HIF-1α. In contrast, under low oxygen conditions, the activity of prolyl hydroxylases is inhibited, leading to an upregulation of HIF-1α expression. The expression of HIF-1α is regulated by several members of the USP family, with different regulatory mechanisms at play. In colorectal cancer, Kateryna Kubaichuk et al. found that USP10 indirectly promotes the synthesis of HIF-1α protein by activating the mTOR/S6K signaling pathway (58). In hepatocellular carcinoma, Qiaonan Shan et al. discovered that phosphorylation of the 1943rd serine residue of MYH9 recruits USP22 to bind with HIF-1α. USP22 then removes the ubiquitin tags from HIF-1α, maintaining its protein stability (59). Furthermore, Mikael Altun et al. found that USP19 can help maintain HIF-1α protein stability by directly interacting with the PAS and bHLH domains at the N-terminal of HIF-1α, without relying on its deubiquitinating activity (60). Notably, USP38’s regulation of HIF-1α is similar to the function of USP22. In non-small cell lung cancer, USP38, upon binding with HIF-1α, removes the K11-linked polyubiquitin chains at Lys 769 of HIF-1α, preventing its proteasomal degradation and thereby maintaining the stability of the HIF-1α protein (61).

The maintenance of HIF-1α protein stability by USP38 has significant clinical implications. Unlike the indirect regulation by USP10 and the non-catalytic binding function of USP19, the classic deubiquitinating activity of USP38 makes it a potential new therapeutic target for non-small cell lung cancer (NSCLC). In the context of increasing resistance to various conventional targeted therapies, the development of USP38-targeted inhibitors for NSCLC could potentially offer more precise and effective tumor treatments.

#### 6.1.7 JAK2/STAT3 signaling pathway

The JAK2/STAT3 signaling pathway is one of the most common intracellular signaling pathways and is essential for the maintenance of normal cellular homeostasis. However, when this pathway is aberrantly activated, STAT3 regulates the transcription and abnormal expression of downstream molecules associated with tumor progression. In the development of malignant tumors, the JAK2/STAT3 signaling pathway primarily participates in the regulation of biological functions such as tumor cell growth, proliferation, migration, invasion, and apoptosis. For example, in nasopharyngeal carcinoma, Yuting Zhan et al. discovered that G3BP1, an RNA-binding protein, directly binds to JAK2 mRNA to promote JAK2 expression and transcription. This further activates the JAK2/STAT3 signaling pathway, leading to the malignant behaviors of nasopharyngeal carcinoma cells, such as increased proliferation, migration, and invasion. Recent studies have shown that most members of the USP family are involved in regulating the aberrant activation of the JAK2/STAT3 signaling pathway in malignant tumors (62). Based on current research, the USPs that positively regulate this pathway include: USP4, USP5, USP8, USP9X, USP14, USP17, USP18, USP21, USP22, USP24, USP28, and USP30. The USPs that negatively regulate this pathway include: USP15 and USP38, while USP7 has a dual regulatory role (63–75). Similar to the negative regulation by USP15, Ting Hu et al. found that overexpression of USP38 in glioma inhibits the aberrant activation of the JAK2/STAT3 signaling pathway, likely through its deubiquitinating activity. Furthermore, related studies have also found that USP38 effectively promotes vemurafenib-mediated inhibition of downstream molecules such as MMP and LAMP, thereby reducing the migration and invasion capabilities of tumor cells. This tumor-suppressing function of USP38 makes it a potential novel target for targeted therapy in glioma (76).

It is noteworthy that, in contrast to the positive regulatory role of USP38 in malignancies such as gastric and colorectal cancers, USP38’s suppression of JAK2/STAT3 signaling pathway activation in glioma demonstrates its dual role in tumor regulation. This characteristic makes USP38 a promising candidate for targeted cancer therapy, as the USP38/JAK2/STAT3 signaling axis regulation may be specific to certain malignancies, such as glioma.

#### 6.1.8 PD-L1

PD-L1, as a cell membrane protein, can bind to its receptor PD-1 under physiological conditions, exerting immunosuppressive functions by inhibiting T cell activation and proliferation (77). In the tumor microenvironment, PD-L1 helps tumor cells evade recognition and attack by immune cells, thereby effectively promoting tumor malignancy.

Regarding whether USP38 directly or indirectly regulates PD-L1 expression, this issue was mentioned in the study by Zhiru Wang et al. Based on data obtained from the GEPIA database, the researchers conducted a correlation analysis and found that USP38 is positively correlated with PD-L1 in gastric cancer (78). However, this study did not experimentally validate the specific interaction mechanism between USP38 and PD-L1. It is noteworthy that, similar to USP38, Yu Wang et al. found that USP22 also exhibits a positive correlation with PD-L1 expression. Through Western blot and immunoprecipitation experiments, the researchers discovered that USP22 can directly bind to PD-L1 and remove the ubiquitin chains from multiple lysine residues on PD-L1 through its deubiquitination activity (79). Based on the functional similarities between USP22 and USP38, we hypothesize that USP38 may also directly bind to PD-L1 to exert its deubiquitination function. Furthermore, according to existing research, USP38 has been shown to regulate the stability of HIF-1α protein in non-small cell lung cancer (NSCLC). HIF-1α has been proven to be a target of EZH2 and regulates PD-L1 expression in NSCLC (80), suggesting that USP38 may indirectly regulate PD-L1 expression.

In conclusion, whether USP38 regulates PD-L1 expression directly or indirectly, it plays a role in the tumor immune evasion process. Moreover, the promotion of PD-L1 expression by USP38 contributes to immune evasion by tumor cells, which undoubtedly increases the potential for resistance to PD-L1 inhibitors. Therefore, in the future, we will further experimentally validate the specific regulatory mechanisms of USP38 on immune regulatory factors, such as PD-L1.

#### 6.1.9 Survivin

Survivin, a member of the inhibitor of apoptosis protein (IAP) family, regulates biological processes such as cell proliferation and apoptosis. In malignant tumors, the high expression of Survivin and its regulatory role in tumor growth make it a potential target for cancer therapy (81). Several inhibitors targeting Survivin, including YM155, FL-118, and EM-1421, have been discovered. These inhibitors effectively block tumor progression by targeting and suppressing Survivin expression. In studies exploring the maintenance of Survivin protein stability, USP1 (82), USP2 (83), USP22 (84), USP35 (85), and USP36 (86)have all been shown to remove the polyubiquitin chains attached to Survivin’s amino acid residues through their deubiquitinating activity, preventing its degradation by the proteasome. Similarly, Wei Wang et al. discovered that USP38 also maintains Survivin protein stability by removing the polyubiquitin chains attached to it. This positive regulatory role suggests that USP38 may serve as a potential tumor biomarker involved in regulating tumor cell growth (85).However, current research on the USP38/Survivin signaling axis is limited to breast cancer and hepatocellular carcinoma. Whether this regulatory function occurs in other tumors remains to be further validated through experiments.

#### 6.1.10 FASN

FASN (fatty acid synthase) is a key regulatory molecule in fat metabolism, catalyzing the conversion of acetyl-CoA and malonyl-CoA into palmitic acid. With the increasing focus on tumor microenvironment research in recent years, FASN has been found to play a significant role in tumor growth, proliferation, and immune evasion (87). FASN in the tumor microenvironment is regulated directly or indirectly by several upstream molecules, and this regulation determines the levels of fatty acids and other nutrients in the tumor microenvironment, influencing tumor cell metabolism and growth. For example, in colorectal cancer, long-chain non-coding RNA POU6F2-AS1 promotes the expression of YBX1 by directly binding to it, and YBX1, in turn, binds to the promoter region of FASN to activate its transcription. The aberrant expression of FASN promotes the synthesis of new fatty acids, effectively regulating the tumor microenvironment of colorectal cancer cells (88). Additionally, FASN enhances the immune evasion capability of tumor cells. In hepatocellular carcinoma, Jiao Huang et al. found that palmitic acid synthesized by FASN can bind to the cysteine residues of MHC-I under the induction of DHHC3, leading to the palmitoylation of MHC-I. While MHC-I plays a crucial role in antigen presentation, palmitoylation of MHC-I results in the loss of this immune function, impairing its ability to clear tumors (89). Furthermore, effectively inhibiting FASN expression can significantly block tumor cell growth. In oral squamous cell carcinoma, de Almeida et al. discovered that after treatment with the FASN inhibitor Orlistat, the expression of Cyclin B1, a downstream regulator of the cell cycle, significantly decreased, leading to tumor cell apoptosis (90). This suggests that inhibiting FASN expression may enhance tumor cell sensitivity to chemotherapy drugs.

The stability of FASN protein expression is regulated by various post-translational modifications, such as acetylation, palmitoylation, and ubiquitination. In studies on nasopharyngeal carcinoma radio-sensitivity, Yuting Chen et al. found that the CAT catalytic domain of USP14 can bind to the TE and MAT functional domains on FASN, and through its deubiquitinating activity, prevents FASN from being degraded by the proteasome (91). In studies on the malignant progression of osteosarcoma, VCP recruits USP2 to act on FASN, removing the K48 polyubiquitin chains on FASN to block its degradation. The FASN/USP2/VCP axis effectively promotes autophagy in tumor cells (92). In gastric cancer, researchers found that USP38, similar to USP2 and USP14, also prevents FASN degradation by removing polyubiquitin chains from the amino acid sequence of FASN, with this process catalyzed by thiol groups (93).

Like other members of the USP family, USP38 can regulate FASN protein expression, indicating that USP38 has the potential to indirectly regulate lipid metabolism in the tumor microenvironment. Additionally, since FASN has been shown to be an oncogenic protein, the regulation of FASN by USP38 may not be limited to gastric cancer. It is necessary to validate the activation of the USP38/FASN signaling pathway in more tumor types. Such exploration of tumor universality will provide strong theoretical support for the development of USP38 inhibitors.

### 6.2 upstream regulatory mechanisms

Compared to the downstream regulatory factors and signaling pathways regulated by USP38, research on upstream pathways is relatively scarce. It has only been found that the SENP2 and PIAS1, ADAR, and METTL14 signaling axes participate in the regulation of USP38 expression.

#### 6.2.1 KLF5

KLF5 (Kruppel-like factor 5), a transcription factor containing a zinc finger domain, directly acts on specific sequences in the promoters of target genes to regulate transcription (94). KLF5 has been shown to play an oncogenic role in various malignant tumors by indirectly affecting tumor growth, proliferation, malignant metastasis, and the maintenance of tumor microenvironment homeostasis through the regulation of signaling pathways (95). In pancreatic cancer, KLF5 acts on the TCCCCTTCCC sequence in the promoter of the downstream target BRCA1, promoting transcription and increasing BRCA1 expression levels. High levels of BRCA1 are then recruited to DNA damage sites by γ-H2AX, repairing broken DNA double strands and preventing tumor cell apoptosis. KLF5 can also regulate the expression of downstream tumor-related molecules. Similarly, KLF5 expression is regulated by various upstream factors (96). The maintenance of KLF5 protein homeostasis is regulated by both ubiquitination and deubiquitination processes. Several molecules, such as WWP1 and NEDD4L, promote the degradation of KLF5 through ubiquitination, while others, including USP38, USP3, UCHL1, and ATXN3L, inhibit KLF5 degradation via deubiquitination. In breast cancer, Yingying Wu et al. found that the carboxyl-terminal active region of USP3 (residues 111–521aa) binds to the amino-terminal region of KLF5 (residues 1–200aa) to promote KLF5 expression via deubiquitination (97). Similarly, in lung adenocarcinoma, USP38 also exerts deubiquitination activity on KLF5. Tao Zhang et al. found that through Co-IP experiments and ubiquitination assays, USP38 can bind to KLF5 and remove ubiquitination marks from KLF5 via deubiquitination. To further clarify the specific binding site of USP38 on KLF5, the researchers constructed amino acid sequence deletion mutants of both proteins. The results showed that the amino acid sequence 1–400 of USP38 could directly bind to the 1–200 aa fragment of KLF5 (98). Notably, unlike USP3, the active region of USP38 is located at the amino terminus (1-400aa), indicating that although both USP38 and USP3 bind to the same active region of KLF5, the differences in their respective active regions may lead to differential sensitivity in regulating KLF5 expression.In addition, researchers have found that the regulation of KLF5 expression by USP38 is also influenced by upstream SUMO modification. Results from IP and Western blotting experiments showed that the de-SUMOylase SENP2, after binding with USP38, inhibits USP38’s deubiquitination activity on KLF5. On the other hand, when the SUMOylase PIAS1 binds with USP38, it promotes USP38’s cleavage of the ubiquitin chain on KLF5. These results indicate that the activation of the USP38-KLF5 signaling axis is dynamically regulated by upstream SUMOylation modifications. However, it is worth noting that the study did not further explore how the upstream pathways regulate the expression of SENP2 and PIAS1.

Currently, there is still a lack of systematic quantitative analysis comparing the effects of different USPs on KLF5 expression levels. Given that investigating the differences in USP regulation of KLF5 expression is important for the development of USP inhibitors, more experiments are needed in the future to further validate the differential regulation of KLF5 by various USPs. Furthermore, it is noteworthy that in lung adenocarcinoma, KLF5 not only regulates the KLF5-MLK4-PCK1 signaling axis but also influences drug metabolism pathways associated with Cytochrome P450 enzymes, highlighting the importance and complexity of KLF5 in regulating lung adenocarcinoma development. Since the regulation of tumor-associated signaling pathways by KLF5 is USP38-dependent, USP38 becomes a potential therapeutic target for lung adenocarcinoma, providing a new direction for the precise treatment of lung adenocarcinoma.

#### 6.2.2 ADAR

ADAR (Adenosine Deaminases Acting on RNA) is an enzyme with catalytic activity that primarily catalyzes the deamination of adenosine on single-stranded RNA. Through the process of RNA editing, ADAR converts adenosine (A) to inosine (I), thereby influencing the subsequent gene expression of specific molecules (99). In addition to regulating the physiological functions of normal cells, ADAR has also been shown to play a role in regulating the initiation and progression of several malignant tumors. In both breast cancer and glioblastoma, ADAR regulates tumor progression through the p53 signaling pathway, but it exhibits completely opposite effects in these two cancers. In breast cancer, ADAR suppresses p53 expression, weakening the tumor-suppressive effect of the p53 signaling pathway and promoting breast cancer cell growth and proliferation. However, in glioblastoma, ADAR, upon being recruited by the lncRNA BDNF-AS, forms a functional complex by binding to p53 mRNA. This interaction enhances the stability of p53 mRNA and effectively inhibits tumor cell growth (100, 101). The dual role of ADAR in regulating tumor progression in different cancers suggests that ADAR’s regulation of downstream signaling pathways may be tissue-specific.

To investigate whether ADAR plays a similar regulatory role in esophageal squamous cell carcinoma (ESCC), Junjing Qiao et al. hypothesized that ADAR could serve as a novel biomarker for ESCC based on its RNA editing function (102). Further mechanistic studies confirmed their hypothesis, with Zhong Wu et al. discovering that overexpression of SOX2 activates gene loci with ERV sequences and increases the chromatin accessibility of these regions, which subsequently enhances dsRNA accumulation. Since ADAR can reduce the toxicity caused by dsRNA accumulation, the increased accumulation of dsRNA undoubtedly increases tumor cell dependence on ADAR (103). This dependency effectively demonstrates ADAR’s regulatory role in the malignant progression of ESCC. In recent years, with the ongoing research into ADAR’s regulatory mechanisms, Qingyong Hu et al. discovered that in ESCC, ADAR can indirectly affect the occurrence and development of malignant tumors by regulating the expression of USP38. However, differing from previous research approaches that directly explored USP38 downstream target molecules, the researchers, after referencing the deubiquitination function of USP4, constructed USP38 wild-type and inactive mutant variants. Immunoprecipitation experiments demonstrated that USP38 requires the catalytic activity site C454 to perform its own deubiquitination function. The researchers then conducted IP and co-IP experiments and constructed various mutants of USP38 and ADAR. The results indicated that ADAR specifically binds to the USP domain of USP38 through its own deaminase domain (591–926 aa) and promotes its expression. Therefore, ADAR is considered an enhancer of USP38 expression.

It is important to note that while USP38 has been shown to influence the malignant progression of various tumors through the regulation of tumor-related factors, most research on USP38’s mechanisms has focused on downstream signaling pathways, with insufficient exploration of the upstream regulatory molecules controlling USP38 expression. The discovery of the ADAR-USP38 signaling axis deepens our understanding of USP38’s tumor regulation mechanisms and provides new insights for the further development of USP38-targeted inhibitors.

#### 6.2.3 METTL14

METTL14 is a protein with methyltransferase catalytic activity that regulates various biological processes through m6A modification (105). In recent years, studies have shown that abnormal m6A levels are closely associated with the malignant progression of tumors, and METTL14 can influence the activation of multiple downstream tumor-related signaling pathways by regulating m6A modifications. In colorectal cancer, Xiaoxiang Chen et al. discovered that METTL14 promotes the m6A modification of SOX4 mRNA, leading to a decrease in SOX4 expression. This modification blocks the activation of the EMT and PI3K/Akt pathways, thus inhibiting tumor growth (106). In pancreatic cancer, Min Wang et al. found that METTL14-induced m6A modification is added to the 3’-UTR region of PERP mRNA, effectively inhibiting its transcription and expression. Known as a p53-dependent effector with tumor-suppressing functions, the inhibition of PERP promotes the growth and proliferation of pancreatic cancer cells (107).

METTL14’s protein stability is regulated by various molecular modifications. Zhancheng Zeng et al. found that STUB1, acting as an E3 ubiquitin ligase, promotes the formation of polyubiquitin chains at lysine residues K148, K156, and K162 of METTL14 upon binding, leading to accelerated proteasomal degradation of METTL14 (108). Additionally, Zizhao Yang et al. discovered that NBR1, a selective autophagy receptor protein, induces the entry of METTL14 into autophagosomes for degradation by recognizing and binding METTL14 (109). These findings suggest that STUB1 and NBR1 play important roles in maintaining METTL14’s stability and preventing its overexpression. Interestingly, in contrast to the functions of the aforementioned molecules, USP38 positively regulates METTL14 expression through a deubiquitination mechanism. In bladder cancer, Ji Huang et al. found that METTL14 enhances USP38 mRNA stability by regulating m6A modifications, and USP38, in turn, deubiquitinates METTL14, promoting its expression by removing polyubiquitin chains from METTL14’s amino acid residues (110). The positive feedback loop mediated by USP38 effectively inhibits the malignant progression of bladder cancer, underscoring its important role in the regulatory mechanism of METTL14.

In recent years, various inhibitors targeting METTL3 have been discovered, but the development of METTL14 inhibitors has encountered significant challenges, likely due to the relatively low catalytic activity of METTL14. The discovery of the USP38/METTL14 positive feedback loop regulation mechanism undoubtedly addresses the difficulty in identifying new targets, providing a new direction for inhibitor development. Moreover, targeting both USP38 and METTL14 in therapy could theoretically enhance the inhibition of bladder cancer tumor cells, offering new insights and recommendations for targeted treatment of bladder cancer.

## 7 Current status and prospects of USP38 inhibitors

In the current study, USP38 has been confirmed to play a crucial regulatory role in the occurrence and progression of various malignancies. This finding provides a key foundation for the development of USP38 inhibitors, highlighting their significance in clinical targeted therapies. Therefore, although no USP38 inhibitors have been published so far, by referencing the mechanisms and principles of other popular small-molecule USP inhibitors (such as USP7 inhibitors P5091 and PU7-1), we can still offer ideas and theoretical foundations for future USP38 inhibitor development.

Due to their structural and functional adaptability, broad application scope, and excellent drug-like properties, small-molecule compounds have become one of the most commonly used substances in inhibitor development. P5091 is a trisubstituted thienyl compound, and in 2012, Dharminder Chauhan and colleagues first demonstrated through high-throughput screening that P5091 can act as a specific inhibitor of USP7, effectively inhibiting USP7 expression. In this study, the researchers confirmed the specific mechanism of P5091 in inhibiting USP7 through isopeptidase activity analysis: USP7 removes Ub7 and K48 polyubiquitin chains from target protein amino acid residues, thereby preventing the target protein from being degraded by the proteasomal system. P5091 specifically recognizes the catalytic active site of USP7, hindering USP7’s precise cleavage of specific ubiquitin chains (111). Similarly, USP38 also possesses deubiquitinating activity, removing single ubiquitin or polyubiquitin chains from target proteins. Existing studies show that the catalytic active site of USP38 is C454, which can recognize and bind various ubiquitin chains, including K11 chains on HIF1α, K33 chains on TBK1, K48 chains on JunB, and K63 chains on HDAC3. This reflects the diverse regulatory functions of USP38. Therefore, based on the development strategy of P5091, we can similarly screen known compound libraries for molecules that specifically bind to the catalytic active site of USP38 and effectively inhibit its deubiquitination activity. Subsequently, we can perform activity and specificity tests to select the ideal USP38 inhibitor.

PROTAC (Proteolysis-Targeting Chimera) is a bifunctional small molecule developed based on the ubiquitin-proteasome system and is a product of new precision targeting regulation technology. It consists of a ligand for the target protein, an E3 ubiquitin ligase, and a linker, and, after binding to the specific target protein, activates the intracellular proteasomal system to degrade the target protein (112). In the study by Jingjie Yi and colleagues, based on the catalytic activity of USP7 at C223, the researchers designed and screened PROTAC compounds, including PU7-1. This compound specifically binds to USP7 in triple-negative breast cancer and promotes its degradation by activating the proteasome. The downregulation of USP7 expression significantly inhibits the proliferation and growth of breast cancer cells. Based on these findings and the structure and function of USP38, we propose a design strategy for USP38 inhibitors. By analyzing the 3D structure of USP38 and using high-throughput screening techniques, we can theoretically identify target protein ligands and E3 ligase ligands (such as CRBN, VHL, etc.) and various types of linkers that can bind to the catalytic amino acid residue C545 of USP38. Then, we can perform *in vitro* experiments by introducing different PROTAC compounds into USP38-overexpressing cell lines. After culturing for a certain period, we can use western blotting to assess USP38 protein expression levels in different groups, selecting the compounds with the best inhibitory effects. Finally, after ensuring selectivity and tissue specificity, we can identify USP38 inhibitors suitable for specific tumors and signaling pathways (113).

In summary, the development of USP38 inhibitors can be achieved through various technical approaches. However, it is important to note that under the influence of various factors, such as the tumor microenvironment, cell type, and genetic background, the regulatory role of USP38 in malignancies may exhibit tissue specificity. Therefore, in different malignancies, we need to select appropriate inhibitors based on the characteristics of USP38 substrates and its specific regulatory mechanisms in the tumor, aiming to improve therapeutic response rates and safety while effectively minimizing off-target effects and drug resistance.

## 8 Discussion and prospect

USP38, a key member of the ubiquitin-specific protease (USP) family, exerts critical control over cellular protein homeostasis through its canonical deubiquitinase (DUB) activity. By recognizing and cleaving polyubiquitin chains from target substrates, USP38 modulates protein stability and function, thereby influencing downstream signaling cascades. Within the context of malignant tumors, USP38 has emerged as a significant regulator, impacting fundamental biological processes including proliferation, migration, invasion, and therapeutic resistance via specific substrate targeting. However, a unifying model for USP38’s role in cancer remains elusive. Accumulating evidence points towards a complex, dichotomous function, where USP38 can act as either an oncogene or a tumor suppressor, precluding simple classification. In colorectal cancer, overexpression of USP38 can activate downstream tumor-suppressive signaling pathways by promoting the expression of key regulatory factors such as HMX3 and HDAC3. However, in malignant tumors such as lung adenocarcinoma, overexpression of USP38 does not promote the activation of the aforementioned tumor-suppressive signaling pathways. Instead, it drives the development of malignancies by promoting the expression of oncogenic factors such as KLF5. This selective regulation of USP38 has been validated in various malignancies. Therefore, we hypothesize that USP38 regulation in tumors may exhibit tissue specificity, selectively activating particular regulatory factors or signaling networks in different malignancies, thereby influencing tumorigenesis and progression.

Furthermore, the influence of USP38 extends beyond tumor cell-intrinsic effects into the complex milieu of the tumor microenvironment (TME). Evidence indicates its involvement in modulating hypoxia responses, metabolic reprogramming, immune cell interactions, cytokine/chemokine profiles, angiogenesis, and stromal dynamics. This interplay between USP38 activity and the TME adds another layer of complexity, suggesting that its net effect on tumorigenesis is orchestrated by a dynamic interplay between cell-autonomous functions and microenvironmental cues. Therefore, understanding the dual role of USP38 necessitates dissecting these intricate, context-dependent interactions within specific tumor ecosystems.

Despite burgeoning interest, significant knowledge gaps persist in the USP38 field, limiting a comprehensive understanding and hindering translational efforts. Firstly, while studies span multiple cancer types, mechanistic investigations often lack sufficient depth. Research predominantly focuses on USP38’s regulation of downstream targets, frequently neglecting the upstream signaling pathways and regulatory factors that govern USP38 expression and activity itself. This incomplete view of the regulatory network can confound interpretation and potentially yield conflicting findings. Secondly, a paucity of robust *in vivo* validation, particularly in malignancies like colorectal cancer, limits the physiological relevance of some findings derived primarily from *in vitro* systems. Thirdly, the role of USP38 in therapeutic resistance constitutes a critical yet underexplored area. Although preliminary data, such as the observed sensitization of HCT 116 cells to Oxal/5-FU upon USP38 overexpression, hint at its potential significance, systematic investigation across diverse cancer types and treatment modalities is largely absent. This represents a major research lacuna with direct clinical implications. Lastly, our structural and functional understanding of USP38 remains rudimentary. While Co-immunoprecipitation (Co-IP) experiments confirm interactions, the precise mapping of catalytic sites and the structural basis for substrate recognition are often missing. The intriguing observation that USP38-mediated KLF5 deubiquitination might involve a region outside the canonical USP domain (residues 1-400) further underscores the need for detailed structural biology studies to potentially reveal non-canonical catalytic mechanisms or interaction interfaces. This ambiguity presents limitations for rational drug design and mechanistic dissection.

The modulation of tumor progression and therapeutic sensitivity by USP38 positions it as an attractive, albeit challenging, therapeutic target. The potential clinical value of modulating USP38 activity, particularly in sensitizing tumors to existing therapies, warrants exploration of specific inhibitors. Currently, USP38 inhibitor development remains in its nascent stages. Strategies mirroring those used for other DUBs, including high-throughput screening (HTS), structure-based drug design (SBDD), fragment-based screening (FBS), and covalent inhibitor design targeting catalytic residues or protein-protein interactions (PPIs), are theoretically applicable. However, key challenges encompass achieving high selectivity over other closely related USPs to minimize off-target toxicities, optimizing pharmacokinetic and pharmacodynamic properties, identifying reliable predictive biomarkers for patient stratification, and fully elucidating USP38’s diverse biological functions to anticipate potential on-target liabilities. While the prospects for USP38 inhibitors as monotherapy are uncertain, combination strategies integrating USP38 modulation with chemotherapy, immune checkpoint inhibitors (ICIs), targeted agents, or potentially gene therapies hold promise for synergistic efficacy and deserve thorough investigation. The precedent set by inhibitors targeting other USPs, such as the clinical and preclinical progress with USP7 inhibitors (e.g., P5091 targeting Cys223), provides a valuable conceptual framework and technological insights applicable to USP38 drug discovery efforts. Identifying and targeting specific active sites – whether canonical or potentially non-canonical – remains a central goal for developing potent and selective inhibitors.

In summary, this review synthesizes recent advancements delineating the complex regulatory landscape governed by USP38 in cancer. We have summarized its aberrant expression patterns, highlighted its multifaceted roles in modulating tumor biology and the TME, and detailed the molecular mechanisms underpinning its context-dependent functions. USP38 clearly plays a pivotal role in malignant progression, and targeting its activity represents a promising avenue for novel therapeutic interventions. However, significant challenges persist regarding its intricate structure-function relationships, its precise contribution to drug resistance, and the development of clinically viable inhibitors. Future research must prioritize (1): Deep mechanistic studies elucidating both upstream regulatory inputs and downstream effector pathways in diverse, physiologically relevant models (including *in vivo* systems) (2); Systematic investigation of USP38’s role in resistance to various anti-cancer therapies across multiple tumor types (3); Detailed structural and functional characterization of USP38, including mapping all potential catalytic sites and substrate interaction interfaces; and (4) Rigorous preclinical validation of USP38 inhibitors, both as monotherapy and in combination regimens. Addressing these critical questions will be instrumental in fully understanding USP38’s biology and realizing its translational potential in oncology.
